# Supplementary material for: The causal role of circulating inflammatory markers in osteoporosis: a bidirectional Mendelian randomized study
Source: Front Immunol. 2024 Jul 18;15:1412298. doi: 10.3389/fimmu.2024.1412298 (PMC11291241; doi:10.3389/fimmu.2024.1412298)
Supplement: Supplementary file 1 [file DataSheet_1.zip › supplementary material/OSTEOPOROSIS-CIM/GCST90274837.tsv.gz_forest.pdf]

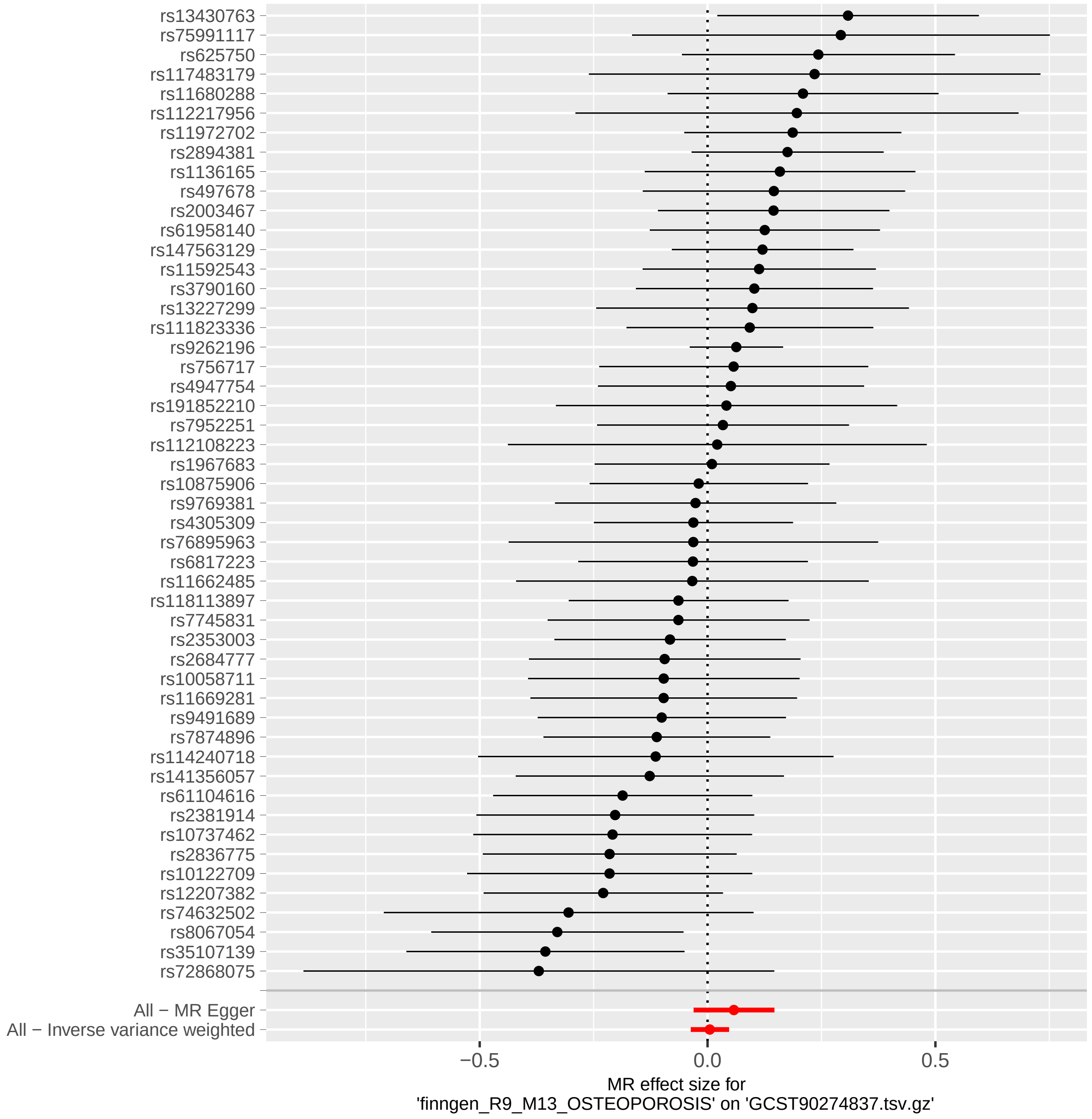
rs13430763
rs75991117
rs625750
rs117483179
rs11680288
rs112217956
rs11972702
rs2894381
rs1136165
rs497678
rs2003467
rs61958140
rs147563129
rs11592543
rs3790160
rs13227299
rs111823336
rs9262196
rs756717
rs4947754
rs191852210
rs7952251
rs112108223
rs1967683
rs10875906
rs9769381
rs4305309
rs76895963
rs6817223
rs11662485
rs118113897
rs7745831
rs2353003
rs2684777
rs10058711
rs11669281
rs9491689
rs7874896
rs114240718
rs141356057
rs61104616
rs2381914
rs10737462
rs2836775
rs10122709
rs12207382
rs74632502
rs8067054
rs35107139
rs72868075
All − MR Egger
All − Inverse variance weighted
−0.5
0.0
0.5
MR effect size for
'finngen_R9_M13_OSTEOPOROSIS' on 'GCST90274837.tsv.gz'
